# Evaluation of the quality of life of individuals with head and neck cancer undergoing surgery and radiotherapy

**DOI:** 10.1590/1807-3107bor-2026.vol40.040

**Published:** 2026-07-10

**Authors:** Lays Ellen Oliveira Menezes, Ana Livia Costa Soares, Giulianna Aparecida Vieira Barreto, Carlos Heli Bezerra Leite, Elyara Soares Veras, Paulo Goberlânio de Barros Silva, José Fernando Bastos de Moura

**Affiliations:** (a) Instituto do Câncer do Ceará, Fortaleza, CE, Brazil.; (b) A. C. Camargo Cancer Center, São Paulo, SP, Brazil

**Keywords:** Head and Neck Neoplasms, Quality of Life, Radiotherapy, Bread

## Abstract

Head and neck cancer (HNC) affects thousands of people worldwide. It is characterized by lesions in the oral cavity, larynx, and pharynx and is often associated with smoking and alcohol consumption. Surgery and radiotherapy improve survival but can cause sequelae that affect daily functioning and quality of life (QoL). This study aimed to assess QoL and functioning in individuals with HNC treated by surgery and radiotherapy. This descriptive, cross-sectional, prospective study enrolled 80 patients between July and September 2024. QoL was measured using the UW-QOL questionnaire, and clinical and sociodemographic data were collected through anamnesis. Statistical analyses were performed using the Friedman–Dunn test for QoL domains, and Fisher's exact test or Pearson's chi-square test for associations. Variables with p < 0.200 were further evaluated by multinomial logistic regression at a 95% confidence level using SPSS v20.0. The participants were predominantly male (70%), with a mean age of 61 years. Most patients (52.5%) reported low QoL; 43.8% reported moderate QoL. The most frequent complaints were fatigue (38.8%) and pain (20%). Low education, pain history, three-dimensional conformal radiation therapy (3D-RT) modality, and previous surgery were significantly associated with reduced QoL (p < 0.05), with low education being the main predictive factor. HNC significantly affected the QoL and functional status of patients. Rehabilitation focusing on physical and emotional support is essential for improving affected domains.

## Introduction

Head and neck cancer (HNC) is a heterogeneous group of neoplasms originating in the oral cavity, pharynx, and larynx, with approximately 200,000 new cases diagnosed annually.^1^ The disease is approximately twice as common in men as in women and is the seventh leading cause of death worldwide.^2^

The principal risk factors for HNC are smoking and excessive alcohol use, which increase disease risk substantially. Additional risks include unprotected sun exposure, human papillomavirus infection, and occupational exposure to carcinogens.

The main therapeutic modalities for HNC include surgery alone or in combination with radiotherapy, chemotherapy, immunotherapy, and targeted therapy. During treatment, patients may experience significant functional impairments that affect swallowing, speech, and quality of life (QoL).^3,4^

Studies have indicated the five-year relative survival rate to be 65% for all cases of HNC, reaching 84% for localized tumors. However, postsurgical scarring and radiotherapy-induced fibrosis can result in several functional changes, the most frequently reported ones being xerostomia, mucositis, fatigue, changes in appearance and voice, difficulty in swallowing, and pain. These side effects can trigger significant emotional responses that affect QoL.^5^

Despite advances in treatment, HNC and its management can significantly compromise functions such as breathing, swallowing, and speech, leading to substantial physical, emotional, and social consequences. Understanding their impact on QoL is crucial for patient care.^6^

The heterogeneity of patients with HNC in terms of tumor location, diversity of surgical techniques, chemoradiotherapy regimens, and individual differences in response to these factors makes it difficult to accurately describe the results associated with different treatment options.^7^

Given the potential of HNC to severely impede speaking, swallowing, and breathing, it is essential to document these impacts. Validated questionnaires are valuable tools for assessing patient experience.

This study aimed to evaluate the QoL and functionality in patients with HNC treated with surgery and radiotherapy.

## Methods

### Study design

This descriptive, quantitative, cross-sectional field research study was conducted through anamnesis and questionnaire administration between July and September 2024 after approval by the Ethics Committee of Hospital Haroldo Juaçaba with opinion number No. 6.917.658.

This study was designed and conducted in accordance with the Strengthening the Reporting of Observational Studies in Epidemiology (STROBE) guidelines.

Patients were assessed individually in a confidential interview at the radiotherapy outpatient clinic, before the start of radiotherapy sessions and after the first consultation with the radiotherapist by a single researcher to avoid operator bias, regardless of whether they had undergone previous surgery.

### Research population: inclusion and exclusion criteria

Patients over 18 years of age with HNC (stages I, II, III, or IV) in the nasopharynx, maxillary sinuses, oropharynx, mouth, and salivary glands were included, in whom radiotherapy or chemoradiotherapy of the head and neck was indicated after tumor resection surgeries at the Haroldo Juaçaba Hospital - Ceará Cancer Institute.

Patients with tumor recurrence or relapse or a previous history of head and neck radiotherapy were excluded. Patients who dropped out of the study or chose not to answer all the questionnaire questions were also excluded.

During questionnaire administration, each patient's mouth opening movement was assessed for trismus using a millimeter ruler.^8–10^

### Clinical outcomes (The University of Washington Quality of Life and Functioning (UW-QOL) Assessment Form)

The University of Washington, Seattle, questionnaire was developed in 1990 by Ernest A. Weymuller Jr., who sought to produce a specific instrument for patients with HNC. It originally contained nine questions (or domains) and has since been changed. Although it has undergone several revisions, eight domains are common to all versions: pain, appearance, activity, recreation, swallowing, chewing, speech, and shoulder function.

The current version (version 4) used in this study was adapted and validated in Brazil by the Department of Head and Neck Surgery and Otorhinolaryngology of the AC Camargo Cancer Hospital. It has 12 questions related to specific functions of the head and neck, as well as activities, recreation, pain, mood, and anxiety. Each question has three to five response categories, each with scores ranging from 0 (worst) to 100 (best), and a composite score is calculated as the average of the 12 domains. It also has a question that allows the patient to choose which domains are the most important to them, along with an open question for the participant to make any comments they deem necessary.^11,12^

The assessment form prepared by the researcher contained the following aspects: name, telephone number, sex, age, occupation, level of education, presence of pain, limited range of motion, smoking, alcohol consumption, limitations in performing activities of daily living, limitations before radiotherapy, comorbidities, medications in use, and quantities.

### Sample size calculation

Considering that Hu et al. (2018)^13^ observed that patients with HNC have a significant reduction in QoL after surgery (93.6% ± 13.1% vs. 81.5% ± 16.6%), it was estimated that 66 patients should be evaluated to obtain a sample that represents the alternative hypothesis of this study with 90% power and 95% confidence (Fleiss method with continuity correction). To account for a potential sample loss, 20% were added, resulting in 80 patients per study group.

### Statistical analysis

QoL domains were reported as means and standard deviations and compared using Friedman and Dunn tests. Clinical data were expressed as absolute frequencies and percentages and were associated with QoL using Fisher's exact or Pearson's chi-square test. Associations with p < 0.200 were subjected to a multinomial logistic regression model. All analyses were performed with a 95% confidence interval in SPSS v20.0 for Windows.

## Results

In this study, 80 patients diagnosed with HNC were evaluated. The sample predominantly comprised men (70%, n = 56). The mean age of the participants was 61.9 ± 12.9 years, ranging from 21 to 88 years. The most prevalent age range was 51–70 years (57.5%, n = 46), followed by patients over 70 years old (23.8%, n = 19) and those up to 50 years old (18.8%, n = 15) (Table 1).

**Table 1:** Data expressed in absolute and percentage frequency form.

Variables		n (%)
Sex		
	Feminine	24 (30.0)
	Masculine	56 (70.0)
Age (61.9 ± 12.9; 21–88) years		
	< 50	15 (18.8)
	51–70	46 (57.5)
	> 70	19 (23.8)
It works		
	No	50 (62.5)
	Yes	30 (37.5)
Education		
	Illiterate	21 (26.3)
	Fundamental	48 (60.0)
	Average	9 (11.3)
	Superior	2 (2.5)
Complaints		
	No	40 (50.0)
	Yes	40 (50.0)
	Fatigue complaints	31 (38.8)
	Complaints of Pain	9 (11.3)
	Complaints others	4 (5.0)
	Trismus	51 (63.8)
	Previous trismus	11 (13.8)
	Pain	16 (20.0)
Smoke		
	Never	7 (8.8)
	Yes, but it stopped	73 (91.3)
	Yes, and it didn't stop	0 (0.0)
Alcoholism		
	Never	16 (20.0)
	Yes, but it stopped	63 (78.8)
	Yes, and it didn't stop	1 (1.3)
	HAS	22 (27.5)
	DM	13 (16.3)
	Other comorbidities	8 (10.0)
	Medications	31 (38.8)
Location		
	Oral cavity	26 (32.5)
	Nasopharynx	5 (6.3)
Continue		
Continuation		
	Larynx	24 (30.0)
	Maxillary/Salivary Gls	8 (10.0)
	Oropharynx	15 (18.8)
	Hypopharynx	2 (2.5)
PostoperativePostoperative surgery		
	No	16 (20.0)
	Yes	64 (80.0)

Data expressed in absolute and percentage frequency form.

Regarding occupation, 37.5% of the patients (n = 30) were employed. The majority had completed only primary education (60%, n= 48), whereas 26.3% (n = 21) were illiterate (Table 1).

Fatigue was the most frequent complaint (38.8%, n = 31), followed by pain (20%, n = 16). In addition, 63.8% (n = 51) of the patients presented with trismus, and 13.8% (n = 11) had this limitation before treatment (Table 1).

Smoking and alcohol consumption before diagnosis were highly prevalent: 91.3% of the patients (n = 73) were smokers before treatment. However, all quit the habit after starting therapy. Only 8.8% (n = 7) of the participants had never smoked. Regarding alcohol consumption, 78.8% (n = 63) reported regular alcohol consumption, whereas 20% (n = 16) had never consumed alcoholic beverages (Table 1).

Systemic arterial hypertension (27.5%, n = 22) and diabetes mellitus (16.3%, n = 13) were the most common comorbidities. In addition, 38.8% (n = 31) of the patients used continuous medication (Table 1).

The tumors were most frequently located in the oral cavity (32.5%, n = 26), followed by the larynx (30.0%, n = 24). Furthermore, 80% (n = 64) of the patients underwent surgery before radiotherapy (Table 1).

Regarding QoL, the shoulder domain presented the highest score (89.66 ± 17.17), followed by pain (82.81 ± 27.44), speech (75.54 ± 25.84), chewing (63.75 ± 33.73), and swallowing (62.61 ± 28.83), which were significantly higher than the other domains evaluated (p < 0.001) (Figure).

**Figure:**
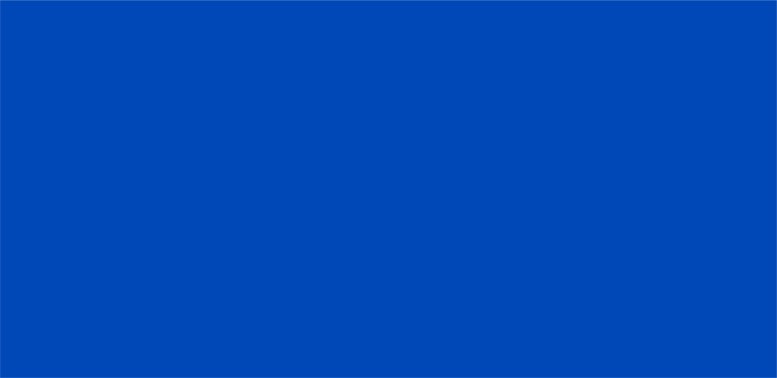
Distribution of QoL scores in patients with HNC, according to the domains evaluated.

Most patients reported a low (52.5%, n = 42) or moderate (43.8%, n = 35) QoL. Only two patients (2.5%) had scores indicative of high QoL, whereas one patient (1.3%) reported very low QoL (Table 2).

**Table 2:** QoL classification and distribution of impacted domains.

Variables		n (%)
QoL		
	From 0 to 25 points: Very low quality of life	1 (1.3)
	From 26 to 50 points: Low quality of life	42 (52.5)
	From 51 to 75 points: Moderate quality of life	35 (43.8)
	From 76 to 100 points: High quality of life	2 (2.5)
Most important problems		
	Activity	51 (63.8)
	Anxiety	48 (60.0)
	Humor	37 (46.3)
	Recreation	29 (36.3)
	Swallowing	23 (28.8)
	Appearance	19 (23.8)
	Palate	8 (10.0)
	Pain	7 (8.8)
	Saliva	7 (8.8)
	Chew	6 (7.5)
	He speaks	5 (6.3)
Compared to the previous month		
	A little worse	6 (7.5)
	More or less the same	5 (6.3)
	A little better	28 (35.0)
	Much better	41 (51.3)
Compared 7d		
	Bad	7 (8.8)
	Average	41 (51.3)
	Good	30 (37.5)
	Very good	2 (2.5)
Considering everything		
	Bad	19 (23.8)
	Average	42 (52.5)
	Good	17 (21.3)
	Very good	2 (2.5)

Among the most relevant problems, limitations in physical activity stood out (63.8%, n = 51), followed by anxiety (60.0%, n = 48) and mood (46.3%, n = 37). Further, 36.3% (n = 29) reported recreational difficulties, and 28.8% (n = 23) reported swallowing difficulties (Table 2).

Compared to the previous month, 51.3% of patients (n = 41) reported significant improvement, whereas 35.0% (n = 28) reported slight improvement. When asked about their condition over the past 7 days, the majority (51.3%, n = 41) rated themselves as "average," whereas 37.5% (n = 30) rated themselves as "good" and 2.5% (n = 2) as "very good." Considering overall QoL, 52.5% (n = 42) described it as "average," 21.3% (n = 17) as "good," and 23.8% (n = 19) as "poor" (Table 2).

When examining the associations between QoL and clinical and demographic variables, a lower QoL was associated with pain complaints (p = 0.044), history of surgery (p = 0.003), and smoking cessation (p = 0.034). In the multivariate analysis, low education level was the main factor related to low QoL (p = 0.032), increasing the odds of low QoL by more than 12 times. Other factors included pain complaints (p = 0.009) (Table 3).

**Table 3:** Bivariate analysis between quality of life and sociodemographic and clinical variables.

Variables		QoL (n [%])		p-Value
		Very low/low	Moderate/high	
Age (years)				
	< 50	6(14.3)	9(23.7)	0.225
	51–70	23(54.8)	23(60.5)	
	> 70	13(31.0)	6(15.8)	
It works				
	No	30(71.4)	20(52.6)	0.083
	Yes	12(28.6)	18(47.4)	
Sex				
	Feminine	14(33.3)	10(26.3)	0.494
	Masculine	28(66.7)	28(73.7)	
Education				
	Illiterate	8(19.0)	13(34.2)	0.088
	Fundamental	27(64.3)	21(55.3)	
	Average	7(16.7)	2(5.3)	
	Superior	0(0.0)	2(5.3)	
Complaints				
	No	21(50.0)	19(50.0)	1
	Yes	21(50.0)	19(50.0)	
Fatigue complaints				
	No	28(66.7)	21(55.3)	0.296
	Yes	14(33.3)	17(44.7)	
	Complaints of pain			
	No	36(85.7)	35(92.1)	0.366
	Yes	6(14.3)	3(7.9)	
Complaints others				
	No	39(92.9)	37(97.4)	0.355
	Yes	3(7.1)	1(2.6)	
Trismus				
	No	13(31.0)	16(42.1)	0.300
	Yes	29(69.0)	22(57.9)	
Previous trismus				
	No	37(88.1)	32(84.2)	0.614
	Yes	5(11.9)	6(15.8)	
Pain				
	No	30(71.4)	34(89.5)*	0.044
	Yes	12(28.6)*	4(10.5)	

* p < 0.05

Fisher's exact or Pearson's chi-square test (n, %).

When QoL was categorized and associated with other characteristics, low QoL was directly associated with a history of pain (p = 0.044) (Table 3), smoking cessation (p = 0.034), and previous surgery (p = 0.003) (Table 4).

**Table 4:** Association between QoL and lifestyle factors, comorbidities, and cancer treatment.

Variable		QoL (n [%])		p-value
		Very low/low	Moderate/high	
Smoke				
	Never	1(2.4)	6(15.8)*	**0.034**
	Yes, but it stopped	41(97.6)*	32(84.2)	
	Yes, and it didn't stop	0(0.0)	0(0.0)	
Alcoholism				
	Never	7(16.7)	9(23.7)	0.484
	Yes, but it stopped	34(81.0)	29(76.3)	
	Yes, and it didn't stop	1(2.4)	0(0.0)	
Systemic arterial hypertension				
	No	33(78.6)	25(65.8)	0.201
	Yes	9(21.4)	13(34.2)	
Diabetes mellitus				
	No	37(88.1)	30(78.9)	0.268
	Yes	5(11.9)	8(21.1)	
Other comorbidities				
	No	39(92.9)	33(86.8)	0.370
	Yes	3(7.1)	5(13.2)	
Medications				
	No	27(64.3)	22(57.9)	0.558
	Yes	15(35.7)	16(42.1)	
Location				
	Oral cavity	14(33.3)	12(31.6)	0.260
	Nasopharynx	2(4.8)	3(7.9)	
	Larynx	14(33.3)	10(26.3)	
	Maxillary/Salivary Gls	2(4.8)	6(15.8)	
	Oropharynx	10(23.8)	5(13.2)	
	Hypopharynx	0(0.0)	2(5.3)	
History of previous surgery				
	No	3(7.1)	13(34.2)	**0.003**
	Yes	39(92.9)	25(65.8)	
Indication for radiotherapy				
	RT-3D	1(2.4)	6 (15.8)	0.061
	IMRT	26(61.9)	24 (63.2)	
	VMAT	15 (35.7)	8 (21.8)	
Prescribed radiotherapy dose				
	< 60 Gy	27 (64.3)	22 (57.9)	0.558
	> 60 Gy	15 (35.7)	16 (42.1)	

* p < 0.05

Fisher's exact or Pearson's chi-square test (n, %).

Multivariate analysis identified that a low QoL in patients was primarily associated with three factors: low education level, pain complaints, and prior surgery. Specifically, patients with low education levels were found to be 20.74 times more likely to report unsatisfactory QoL (95% CI = 1.54–278.49; p = 0.022). Similarly, pain was a significant factor that increased the likelihood of reduced QoL by 18.92 times (95% CI = 2.15–166.61; p = 0.008) (Table 5).

**Table 5:** Multivariate analysis of predictors of low quality of life.

Very Low/Low QoL	p-value	ORa	95%CI
Does not work	0.054	3.27	0.98–10.90
Low education level	0.022	20.74	1.54–278.49
Pain	0.008	18.92	2.15–166.61
Quit smoking	0.067	21.29	0.80–563.66
Previous surgery	0.020	9.78	1.44–66.32
Indication for 3D-RT	0.024	29.96	1.55–578.16
Prescribed radiotherapy dose > 60Gy	0.770	1.19	0.36–3.91

p < 0.05, multinomial logistic regression. ORa = adjusted odds ratio; 95%CI = 95% confidence interval of ORa.

Furthermore, patients who had required prior surgery, reflecting a more severe condition or more invasive treatment, were 9.78 times more likely to report low QoL (95% CI = 1.44–66.32; p = 0.020), and those indicated for three-dimensional conformal radiation therapy (3D-RT) were 29.96 times more likely to report low QoL (95% CI = 1.55–578.16) (Table 5).

## Discussion

The results of this study demonstrate that HNC has a significant impact on patient QoL and functionality, with pain, low education level, and history of previous surgery being predictors of worse QoL.

The predominance of male patients (70%) and those aged 51–70 years is in line with previous studies that associated HNC with risk factors, such as smoking and alcohol consumption, which are most common among men in this age group^13^. Furthermore, the study revealed that 68% of the patients were smokers or ex-smokers, whereas 55% reported frequent alcohol consumption.

A low educational level was shown to predict lower QoL, increasing the probability of a negative evaluation by more than 12 times. This may be related to the difficulty these patients have in understanding medical advice and adhering to treatment, indicating a direct association between educational level and health outcomes.^14^

This reality is even more worrying in the context of the state of Ceará, where the study was conducted, given that the region has one of the highest illiteracy rates in Brazil according to data from the Brazilian Institute of Geography and Statistics (IBGE). This regional characteristic reinforces the need for public policies aimed at health education and simplification of medical information so that patients can actively participate in managing their condition.^15^

Lower scores in the activity, recreation, and anxiety domains (p < 0.001) reflected the physical and emotional challenges faced by these patients. These data are consistent with those of studies indicating that physical and recreational activity limitations can lead to social isolation and reduced emotional well-being.^16^

Meanwhile, domains such as pain (82.81 ± 27.44) and speech (75.54 ± 25.84) obtained higher scores, indicating better functional preservation in some respects. However, these results do not deny the need for interventions, especially given that pain was associated with lower QoL in multivariate analyses, increasing the risk of negative evaluation by 9.39 times (p = 0.009).

Another relevant point is the shoulder domain, which had one of the highest scores. Despite this, its inclusion in the QoL questionnaire for patients with HNC is justified, given the frequent involvement of the brachial plexus during cervical region surgeries and treatments such as neck dissection. Changes in the shoulder, such as pain or movement limitations, may be due to direct or secondary damage, including radiotherapy-induced fibrosis or nerve impairment, affecting functionality and the ability to perform daily activities. Targeted physiotherapy monitoring is essential to minimize sequelae and promote functional rehabilitation.^17^

The relationship between fatigue and pain is also noteworthy, especially according to the National Comprehensive Cancer Network (NCCN) 2024 guidelines. These guidelines indicate that cancer-related fatigue is multifactorial and often exacerbated by inadequately controlled pain. The interaction between fatigue and pain creates a vicious cycle in which pain intensifies fatigue. In contrast, fatigue reduces a patient's ability to manage pain, compromising their functionality and QoL. Integrated approaches such as effective pain control and multidisciplinary support are essential to break this cycle and improve the clinical and emotional outcomes.^18^

The prevalence of tumors in the oral cavity and larynx, the most common sites for HNC, corresponds with research associating these regions with risk factors such as smoking and alcohol consumption. These behaviors increase susceptibility to carcinogens that damage epithelial cells in the upper aerodigestive tract, which are the sites where these tumors typically develop.^19^

Another relevant factor was a history of surgery, which was associated with worse QoL. Surgery for HNC is highly invasive and can cause significant functional and aesthetic sequelae, affecting a patient's self-image, social interaction, and functional capacity. Research has shown that these changes have a lasting impact on QoL, highlighting the need for rehabilitation support for these patients.^20^

Patients who had undergone previous surgeries also presented with a reduction in QoL due to functional and aesthetic sequelae resulting from the procedure. The literature reinforces that surgery for HNC can result in significant limitations, affecting the ability to eat and speak, and impacting patients’ social integration and emotional well-being.^21^ In addition, data comparisons demonstrated that psychosocial support and treatment continuity strategies should be prioritized to achieve more comprehensive and patient-centered care.

These findings highlight the importance of physiotherapy, especially for patients undergoing surgery who present with a significant reduction in QoL. Previous studies have shown that physiotherapy is crucial for improving functionality, such as neck mobility and mouth opening, which are frequently compromised after surgeries and radiotherapy.^22^ The need for rehabilitation interventions was reinforced by the high prevalence of trismus (63.8%), a condition frequently associated with HNC treatment that directly affects the ability to chew and speak.^23^ Implementation of specific protocols to minimize these sequelae should be prioritized.

Anxiety and mood changes indicate that HNC affects not only the body but also the emotional state of individuals. A cancer diagnosis often carries a heavy emotional burden, which can be amplified by uncertainties related to treatment and prognosis.^15^

These data highlight the importance of adequate psychosocial support, which is integral to patient care. Interventions that aim to provide emotional support such as psychological therapy and support groups can be instrumental in helping patients cope with anxiety and promote a more positive state of mental well-being.

A recent systematic review, published in 2021, demonstrated that when comparing intensity-modulated radiation therapy (IMRT) with 3D radiotherapy, considering both acute and late toxicity, IMRT showed lower toxicity than that of 3D radiotherapy—toxicity that directly affects patient QoL—which corroborates the findings of the present study, in which 3D radiotherapy was associated with a worse QoL for patients undergoing radiotherapy for HNC.^24^

Difficulties in oral and recreational functions are also a concern. The inability to fully participate in recreational activities reflects physical limitations and significantly affects social life and QoL. Fun is a vital component of social life and emotional well-being. Difficulties in this area can lead to isolation and decreased life satisfaction.^25^

This study was limited by its small sample size, which may not reflect the entire patient population with HNC. Furthermore, among the 80 patients, there were several types of HNC, including pharyngeal, laryngeal, and oral cancer. Additionally, the questionnaires were administered directly to the patients, limiting the subjectivity of the responses. However, a sample size calculation was performed to validate the sample, which partially mitigated this limitation.

Most patients with head and neck tumors experience a low QoL before initiating radiotherapy. The most affected areas were activities, recreation, and anxiety. The main risk factors were the presence of pain, having undergone tumor resection surgery, and low educational attainment. Therefore, these patient groups require more attention and emotional support even before initiating head and neck radiotherapy.

## Data Availability

The authors declare that all data generated or analyzed during this study are included in this published article.
